# Comparative Evaluation of Radiochemical and Biological Properties of ^131^I- and [^99m^Tc]Tc(CO)_3_-Labeled RGD Analogues Planned to Interact with the α_v_β_3_ Integrin Expressed in Glioblastoma

**DOI:** 10.3390/ph15020116

**Published:** 2022-01-18

**Authors:** Danielle V. Sobral, Leonardo L. Fuscaldi, Ana Claudia R. Durante, Fernanda F. Mendonça, Larissa R. de Oliveira, Ana Cláudia C. Miranda, Jorge Mejia, Wagner R. Montor, Marycel F. de Barboza, Luciana Malavolta

**Affiliations:** 1 Department of Physiological Sciences, Santa Casa de Sao Paulo School of Medical Sciences, Sao Paulo 01221-020, Brazil; danielle_sobral@hotmail.com (D.V.S.); leonardo.fuscaldi@hotmail.com (L.L.F.); fernandaferreiramendonca@hotmail.com (F.F.M.); larissa.rolim26@gmail.com (L.R.d.O.); wagner.santacasa@gmail.com (W.R.M.); 2 Hospital Israelita Albert Einstein, Sao Paulo 05652-900, Brazil; aninhapharma30@gmail.com (A.C.R.D.); ana.miranda@einstein.br (A.C.C.M.); jorge.mecabeza@einstein.br (J.M.); marycel.barboza@einstein.br (M.F.d.B.)

**Keywords:** glioblastoma, glioma cells, α_v_β_3_ integrin receptor, radiolabeled peptides, RGD-analogues

## Abstract

Radiolabeled peptides with high specificity for overexpressed receptors in tumor cells hold great promise for diagnostic and therapeutic applications. In this work, we aimed at comparing the radiolabeling efficiency and biological properties of two different RGD analogs: GRGDYV and GRGDHV, labeled with iodine-131 (^131^I) and technetium-99m-tricarbonyl complex [^99m^Tc][Tc(CO)_3_]^+^. Additionally, we evaluated their interaction with the α_v_β_3_ integrin molecule, overexpressed in a wide variety of tumors, including glioblastoma. Both peptides were chemically synthesized, purified and radiolabeled with ^131^I and [^99m^Tc][Tc(CO)_3_]^+^ using the chloramine-T and tricarbonyl methodologies, respectively. The stability, binding to serum proteins and partition coefficient were evaluated for both radioconjugates. In addition, the binding and internalization of radiopeptides to rat C6 glioblastoma cells and rat brain homogenates from normal animals and a glioblastoma-induced model were assessed. Finally, ex vivo biodistribution studies were carried out. Radiochemical yields between 95–98% were reached for both peptides under optimized radiolabeling conditions. Both peptides were stable for up to 24 h in saline solution and in human serum. In addition, the radiopeptides have hydrophilic characteristics and a percentage of binding to serum proteins around 35% and 50% for the [^131^I]I-GRGDYV and [^99m^Tc]Tc(CO)_3_-GRGDHV fragments, respectively. Radiopeptides showed the capacity of binding and internalization both in cell culture (C6) and rat brain homogenates. Biodistribution studies corroborated the results obtained with brain homogenates and confirmed the different binding characteristics due to the exchange of radionuclides and the presence of the tricarbonyl complex. Thereby, the results showed that both radiopeptides might be considered for future clinical applications.

## 1. Introduction

Radiopeptides in Nuclear Medicine are considered the new generation of biologically active tools, mainly because some of them are key regulators in many physiological responses. Furthermore, they have a great potential to bind specific targets, due to the fact that tumor cells overexpress receptors with high affinity to specific peptides [1,2]. Because of this specificity, the great majority of currently used radiopeptides have been used as diagnostic tracers, including peptides radiolabeled with ^123^I, ^99m^Tc, ^18^F, and ^68^Ga to obtain Single Photon Emission Computed Tomography (SPECT) and Positron Emission Tomography (PET) images of tumors and their possible metastases [3,4]. However, despite the inferior image quality, SPECT radionuclides have wider application in Nuclear Medicine due to the simpler and better-known imaging technology, as well as lower financial requirements, if compared to the PET radioisotopes technique [5].

The use of radionuclides in research is fundamental for the understanding of several mechanisms which depend on high resolution and sensitivity for detection. Molecular oncology studies benefit greatly from such applications [6]. 

Angiogenesis is essential for tumor growth and metastasis [7]. Integrins, which regulate many biological processes, such as cell migration, invasion, adhesion, apoptosis, growth, and differentiation, also play a substantial role in metastasis, as well as in tumor angiogenesis. Many angiogenic inhibitors have been developed in recent years as new therapeutic agents for cancer treatment [3,8,9,10,11].

Among the integrins, the α_v_β_3_ has been the most extensively studied one as it is highly expressed in many solid tumors including glioblastoma, and its expression levels are well correlated with the potential for tumor metastases and aggressiveness [12,13]. Most of the integrin-targeted imaging tracers are based on the Arginine-Glycine-Aspartate (RGD) motif, due to its elevated affinity and specificity for the α_v_β_3_ integrin. In the last decade, many radiolabeled RGD peptide antagonists have been studied as α_v_β_3_ integrin-targeted radiotracers to assess angiogenesis inhibition effectiveness in many tumor types [3,14,15,16].

**Figure 1:**
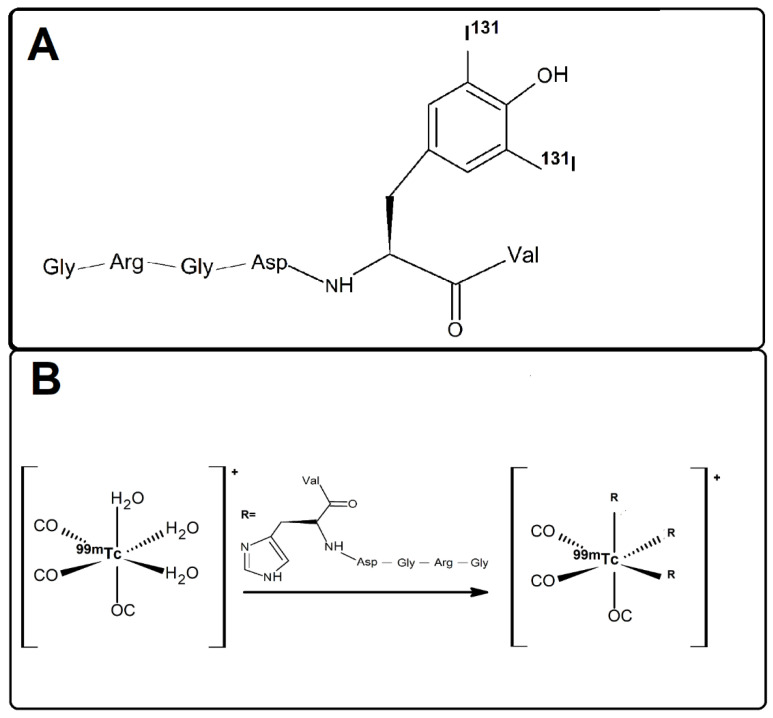
Molecular complexes of (**A**) [^131^I]I-GRGDYV and (**B**) [^99m^Tc]Tc(CO)_3_-GRGDHV. The molecules were drawn using the ACD/Labs 2020 software (File Version C15E41, Build 117352, 2020), adapted from [18,19].

In recent work, [^68^Ga]Ga-labeled peptides (e.g., DOTA-c(RGDfK), NODAGA-c(RGDyK), NODAGA-c(RGDyK)_2_ and DOTA-substance P) were preclinically evaluated and the in vivo behavior was compared with two clinically established [^18^F]F-based tumor radiotracers ([^18^F]-FDG and [^18^F]-FLT) in a mouse xenograft model of human glioblastoma [17]. On the other hand, Vats and co-workers demonstrated the influence of incorporation of technetium-99m-tricarbonyl complex ([^99m^Tc][Tc(CO)_3_]^+^) at the N- and C-terminal portion of Asparagine-Glycine-Arginine (NGR) peptide in order to assess the effect of the introduction of this radiometal at two terminals on the CD13 receptor binding [5]. In the present study, we describe the radiometalation of an RGD-containing peptide, GRGDHV, with a [^99m^Tc][Tc(CO)_3_]^+^ metal fragment, and the radioiodination of another RGD-containing fragment, GRGDYV, with ^131^I (Figure 1). In the former, the three water molecules of the [^99m^Tc][Tc(H_2_O)_3_(CO)_3_]^+^ complex can be replaced by the histidine imidazole ring of the peptide and form a thermodynamically and kinetically stable complex. In the latter, the aromatic ring of the tyrosine was substituted with ^131^I atoms.

It is important to note that radiolabeling studies of RGD-containing peptides are already quite common, and the interaction of these fragments with the α_v_β_3_ integrin molecule is already well described in the literature. However, this is the first time that the influence of the most used radionuclides (^131^I and ^99m^Tc) in the new proposed fragments was compared, regarding radiochemical performance and biological properties.

Thus, the objective of this study was to evaluate the radiosynthesis and biological properties of two RGD-containing peptides using the ^131^I radionuclide (Figure 1A) and the [^99m^Tc][Tc(CO)_3_]^+^ complex (Figure 1B), for the peptides containing the tyrosine (GRGDYV) and histidine (GRGDHV) amino acids, respectively. 

## 2. Results and Discussion

### 2.1. Peptide Synthesis

**Figure 2:**
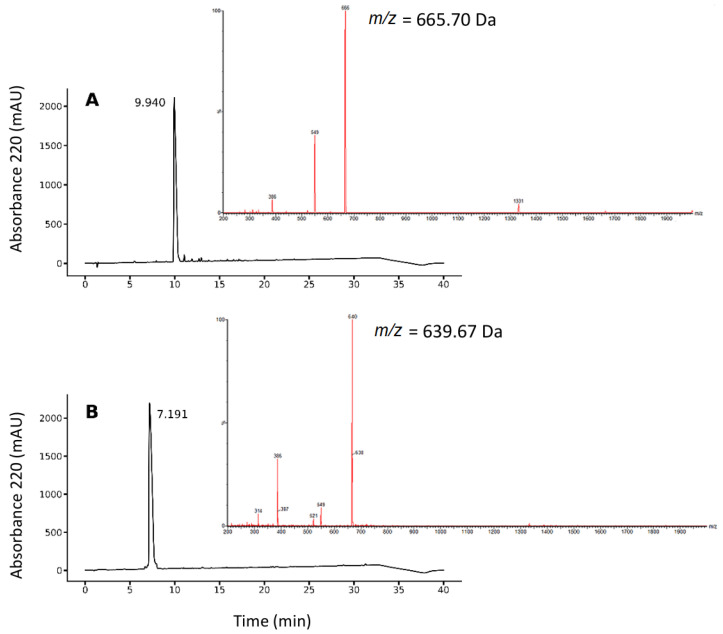
Chromatographic and mass spectrometric profiles for the RGD analogues (**A**) GRGDYV and (**B**) GRGDHV.

The GRGDYV and GRGDHV fragments were efficiently synthesized by solid-phase peptide synthesis, purified by Reversed Phase-High Performance Liquid Chromatography (RP-HPLC), and analyzed by mass spectrometry. In Figure 2, the chromatographic and mass spectrometric profiles are shown, for the GRGDYV (Figure 2A) and GRGDHV (Figure 2B) fragments. The final yield of the synthesis was near 70% for both peptides, whose theoretical molecular weights of 665.70 g·mol^−1^ for GRGDYV and 639.67 g·mol^−1^ for GRGDHV were experimentally confirmed.

### 2.2. Radiolabeling of Peptide Fragments

The chloramine T method was used to radiolabel the GRGDYV fragment with ^131^I [19,20]. The aromatic ring of tyrosine was substituted with iodine atoms by a classic method of radioiodination that involves a slight exchange of nucleophilic halogen, through a reaction that takes place on the surface of the oxidizing agent [18]. It is essential to emphasize that ^123^I and ^131^I are chemically equivalent, and the use of ^131^I in this study was based on its greater availability, lower cost and longer half-life (~8 d).

In turn, the tricarbonyl method was used to radiolabel the GRGDHV peptide with ^99m^Tc [19]. This radioisotope is the most widely used in Nuclear Medicine because of its many advantageous features: (i) easy availability through a ^99^Mo/^99m^Tc generator; (ii) low cost; (iii) emission of low energy gamma rays (~141 KeV), which is compatible with gamma cameras and so, it is commonly used in clinical diagnostics. However, it is important to mention that the tricarbonyl method is not the most usual in the clinics and it was used here for a comparative research proposal.

Radiochemical yield was evaluated by thin layer chromatography (TLC). For [^131^I]I-GRGDYV, an appropriate assessment was performed using Whatmann 3 MM chromatographic paper and MeOH/H_2_O (95:5), while for [^99m^Tc]Tc(CO)_3_-GRGDHV, the best analysis was obtained using thin layer chromatographic-silica gel on aluminum (TLC-SG(Al)) strip and ACN/H_2_O (95:5). [^99m^Tc][Tc(CO)_3_]^+^ complex was evaluated using TLC-SG(Al) strip and 0.9% NaCl.

**Figure 3:**
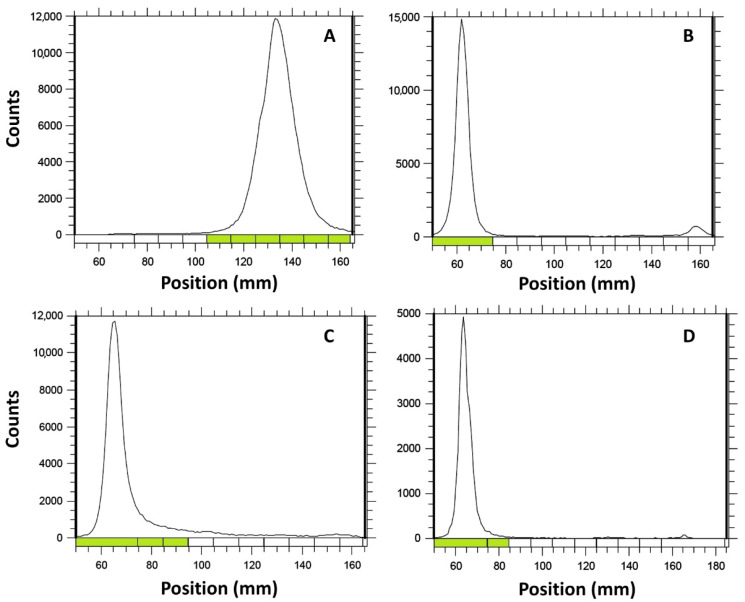
Radiochemical yield evaluated by thin layer chromatography (TLC): (**A**) [^131^I]NaI; (**B**) [^131^I]I-GRGDYV; (**C**) [^99m^Tc][Tc(CO)_3_]^+^; (**D**) [^99m^Tc]Tc(CO)_3_-GRGDHV.

The chromatograms showed that both radiolabeled peptides and the tricarbonyl remain in the origin, with a retention factor (R_f_) of 0.1–0.2, while the [^131^I]NaI migrates with the solvent front (R_f_ = 0.8 − 0.9) (Figure 3). Although the [^99m^Tc]Tc(CO)_3_-GRGDHV and [^99m^Tc][Tc(CO)_3_]^+^ showed similar R_f_, it is important to highlight that the radiochromatograms were obtained in different mobile phases, ACN/H_2_O (95:5) and 0.9% NaCl, respectively. Chromatographic yields of 96.70 ± 0.72% and 98.30 ± 0.17% were obtained for [^131^I]I-GRGDYV (Figure 3B) and [^99m^Tc]Tc(CO)_3_-GRGDHV (Figure 3D), respectively (*n* = 10). In general, radiopharmaceuticals must present high radiochemical purity (>90%) [21]. Figure 3A,C show the chromatographic profiles of [^131^I]NaI and [^99m^Tc]Tc(CO)_3_^−^, respectively.

**Figure 4:**
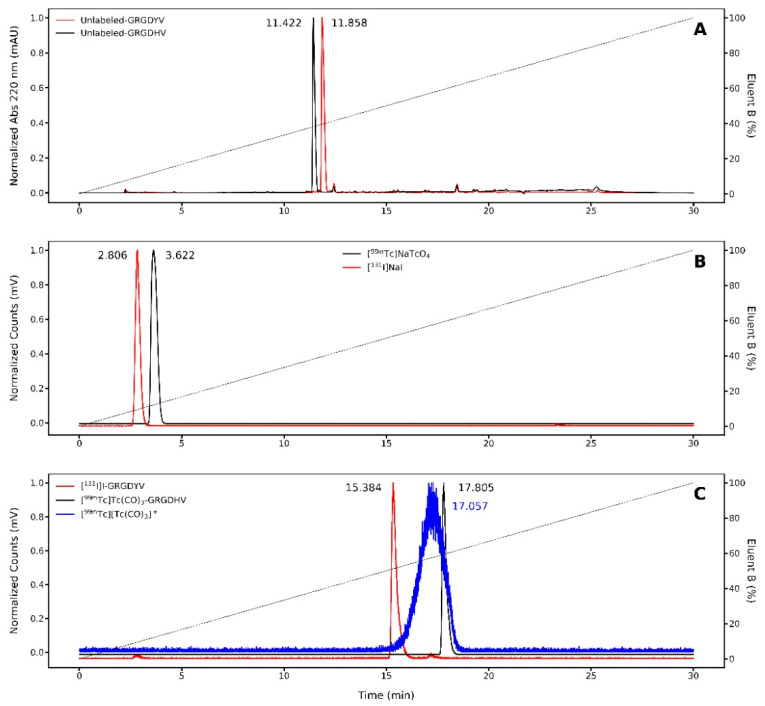
Radiochemical purity evaluated by Reversed Phase-High Performance Liquid Chromatography (RP-HPLC). Chromatograms of (**A**) non-labeled peptides; radiochromatograms of (**B**) [^131^I]NaI, [^99m^Tc]NaTcO_4_, (**C**) radiopeptides and [^99m^Tc][Tc(H_2_O)_3_(CO)_3_]^+^. Each peptide was separately injected.

In order to verify the TLC results, RP-HPLC was also used to determine radiochemical purities of [^131^I]I-GRGDYV and [^99m^Tc]Tc(CO)_3_-GRGDHV (Figure 4). First, the non-labeled peptides were analyzed, displaying retention times (R_t_) of 11.85 and 11.42 min, respectively (Figure 4A). Then, free ^131^I and ^99m^Tc were also evaluated, presenting R_t_ of ~3 min (Figure 4B). Finally, the radiolabeled peptides, [^131^I]I-GRGDYV and [^99m^Tc]Tc(CO)_3_-GRGDHV, presented R_t_ of 15.38 and 17.80 min, respectively (Figure 4C). It is important to mention that a slight time delay between UV and activity signals is caused by the serial arrangement of the detectors, which implies an increase in the R_t_ values of the radiopeptides (24). Beyond that, the [^99m^Tc]Tc(CO)_3_-GRGDHV becomes much more hydrophobic, due to the presence of the [^99m^Tc][Tc(CO)_3_]^+^ complex, and then the R_t_ is longer.

Consequently, RP-HPLC results are in agreement with TLC data and confirmed the success of the radiolabeling, highlighting the importance of the latter for radiochemical yield analysis of labeled peptides in the clinical routine, given that it is a fast, proven and easy-to-perform method [22].

The results demonstrated in Figure 3 and Figure 4 show that both peptides were effectively radiolabeled (>95%), by the respective methods.

### 2.3. In Vitro Evaluation of the Stability of the Radiolabeled Peptides

**Figure 5:**
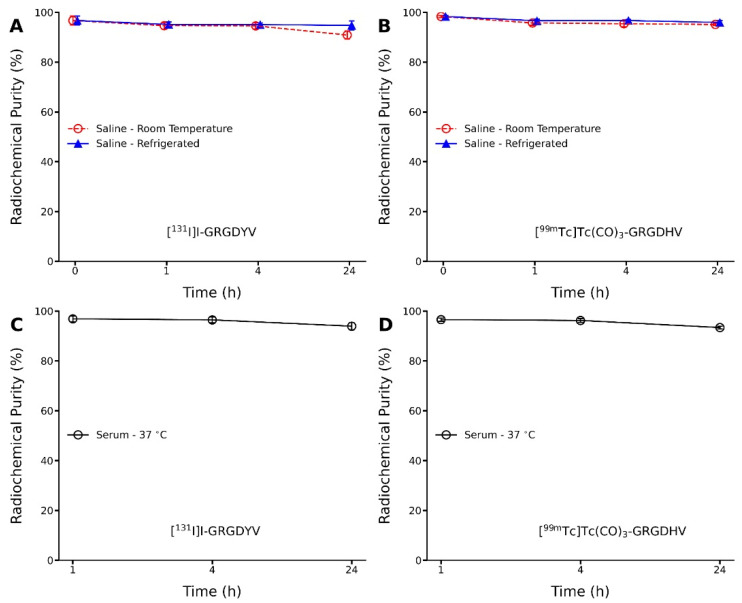
Stability of the radiopeptides up to 24 h (**A**,**B**) in saline solution and **(C**,**D)** in human serum. Data are expressed as ‘mean ± SD’. No significant differences were observed within time (*p* > 0.05).

Stability of both [^131^I]I- and [^99m^Tc]Tc(CO)_3_-peptides was evaluated in saline solution, at room temperature and under refrigeration (4–8 °C), and in serum, at 37 °C, by TLC (Figure 5A–D).

The results showed maintenance of the radiochemical purity up to 24 h for both radiopeptides in saline solution, kept either at room temperature or under refrigeration. Furthermore, the stability of the radiolabeled peptide molecules was also analyzed in human serum at 37 °C for up to 24 h. The results also showed high stability with the radiochemical purity >94%. Therefore, the radionuclides (^131^I or [^99m^Tc][Tc(CO)_3_]^+^ complex) did not influence the radiochemical integrity of peptides in saline nor in human serum.

It is worth mentioning that the stability of radiolabeled peptides may imply a greater fraction of intact radiopeptide available at the target, enlarging the binding likelihood. Therefore, the stability of radiopeptides is an important and decisive parameter for the success of a new tracer.

### 2.4. Partition Coefficient (P) Evaluation

The lipophilicity of the radiolabeled peptides was determined based on their corresponding distribution between n-octanol and water. Mean values of the logarithm of the partition coefficient (Log P) were −3.11 ± 0.49 and −1.13 ± 0.14 (*n* = 6) for [^131^I]I-GRGDYV and [^99m^Tc]Tc(CO)_3_-GRGDHV, respectively. Although both values are within the hydrophilic range (negative values), the presence of [^99m^Tc][Tc(CO)_3_]^+^ precursor in the [^99m^Tc]Tc(CO)_3_-GRGDHV made this peptide less hydrophilic than the [^131^I]I-GRGDYV. The hydrophilic character is associated with renal excretion, which can be favorable for biodistribution and imaging studies. Conversely, lipophilic compounds are extensively reabsorbed and return to the systemic circulation. Elimination of radiolabeled compounds is of critical importance as removal of these molecular complexes from the body allows for improvement of the target to non-target ratio and then, better imaging contrast [23].

### 2.5. Serum Protein Binding

The serum protein binding (SPB) percentages were 34.99 ± 2.07% and 49.97 ± 0.01% (*n* = 6) for [^131^I]I-GRGDYV and [^99m^Tc]Tc(CO)_3_-GRGDHV, respectively. These results corroborate the P data [24]. The [^99m^Tc]Tc(CO)_3_-GRGDHV proved to be less hydrophilic and, in consequence, presented an approximately 15% higher SPB, due to the presence of the tricarbonyl group attached to the peptide fragment. However, in both cases, at least 50% of the radiopeptides are free to reach the target of interest. Although this approach is technically appropriate to estimate SPB and has been described in the literature [25,26,27], there is the possibility of peptide precipitation within the proteins.

### 2.6. Binding and Internalization Analysis of the Radiolabeled Peptides in C6 Tumorigenic Cells

The binding and internalization analysis of the radiopeptides in C6 tumorigenic cells were performed in vitro at 1, 4, and 24 h of incubation. 

**Figure 6:**
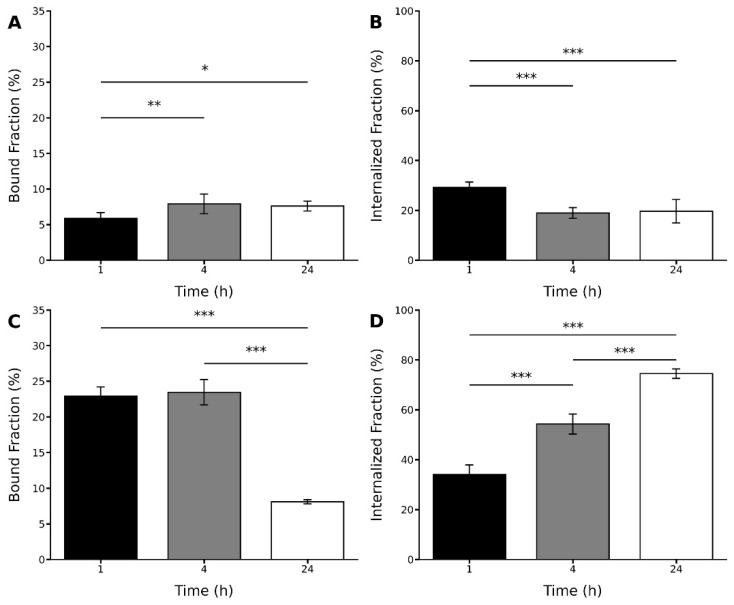
Bound and internalized fractions (%) of radiopeptides in C6 tumorigenic cells: (**A**,**B**) [^131^I]I-GRGDYV and (**C**,**D**) [^99m^Tc]Tc(CO)_3_-GRGDHV. Data are expressed as ‘mean ± SD’. *p* < 0.001 (***), *p* < 0.01 (**) and *p* < 0.05 (*).

The radiopeptides displayed distinct binding and internalization profiles (Figure 6). For the [^131^I]I-GRGDYV, bound and internalized fractions of ~5% and ~29% were obtained after 1 h. At 24 h, binding increased to ~7%, but the internalization decreased to ~19%. In the case of the [^99m^Tc]Tc(CO)_3_-GRGDHV, higher bound and internalized fractions were obtained after 1 h of incubation (~22% and ~34%, respectively). However, after 24 h, a lower bound fraction (~8%) and a higher internalized fraction (~74%) were observed.

These results suggest that the replacement of amino acids and the peptide labeling with different radioisotopes were able to modify the binding and internalization properties of these radiopeptides to glioblastoma-related tumorigenic cells. For instance, for the [^131^I]I-GRGDYV, mean values of 7.91% ± 1.37 and 18.92% ± 2.15 for bound and internalized fractions were obtained after 4 h of incubation (Figure 6A,B). On the other hand, for the [^99m^Tc]Tc(CO)_3_-GRGDHV, bound and internalized percentages were higher (23.47% ± 1.78 and 54.24% ± 3.99, respectively) after 4 h (Figure 6C,D). Thus, in general for this peptide, higher binding and internalization percentages were observed compared to the ^131^I-labeled peptide. The internalization process increases with time for [^99m^Tc]Tc(CO)_3_-GRGDHV. 

### 2.7. Binding and Internalization Studies of the Radiolabeled Peptides in Rat Brain Homogenates

In order to evaluate the relationship between the binding and internalization values observed for C6 cells and the expression of the α_v_β_3_ integrin receptors, the radiopeptides were incubated in brain homogenates from normal rats and a C6-induced glioblastoma model.

**Figure 7:**
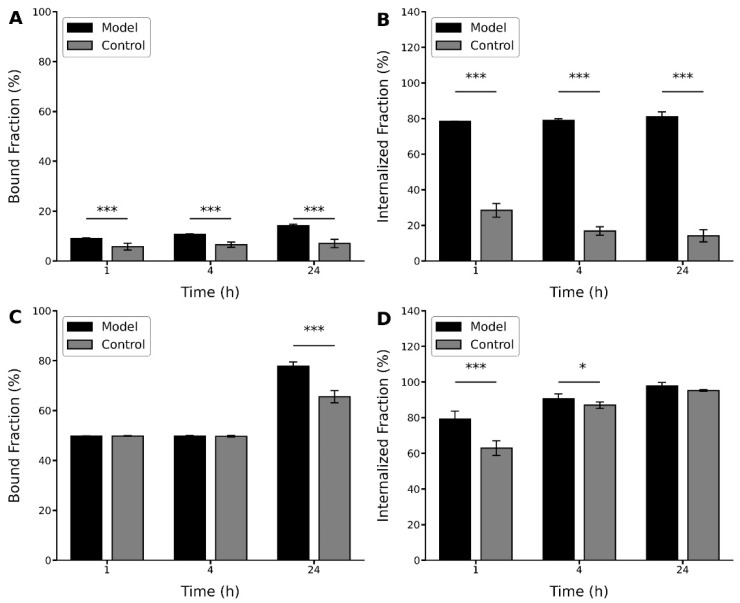
Bound and internalized fractions (%) of radiolabeled peptides in tumor-bearing glioblastoma brain homogenate (model) and normal (control) rats: (**A**,**B**) [^131^I]I-GRGDYV and (**C**,**D**) [^99m^Tc]Tc(CO)_3_-GRGDHV. Data are expressed as ‘mean ± SD’. *p* < 0.001 (***) and *p* < 0.05 (*).

In this study, although different profiles were shown by the peptides, in both cases, higher values were observed for the binding and internalization percentages for the brain homogenates of the glioblastoma model (Figure 7). In this case, the [^131^I]I-GRGDYV showed bound and internalized fractions of ~9% and ~78%, respectively, after 1 h. After 24 h, the binding and internalization percentages increased to ~14% and ~81%, respectively (Figure 7A,B). For the [^99m^Tc]Tc(CO)_3_-GRGDHV (Figure 7C,D), higher bound and internalized fractions were observed in the first hour (~50% and ~79%, respectively) with an increase after 24 h (~78% and ~98%, respectively), corroborating the aforementioned results for C6 cell culture, that showed a higher percentage of bound and internalized fractions for the [^99m^Tc]Tc(CO)_3_-GRGDHV peptide. However, when normal rat brain homogenates were used, the [^131^I]I-GRGDYV peptide showed bound and internalized fractions of only ~6% and ~28% at 1 h, and ~7% and ~14% at 24 h, respectively. In the case of the [^99m^Tc]Tc-(CO)_3_-GRGDHV peptide, the binding and internalization values were ~50% and ~63%, at 1 h and ~65% and ~87% at 24 h, respectively. 

In this work, the whole brain was used for the preparation of normal and tumoral homogenates. Thus, it is noteworthy that larger differences could be observed if only the tumor formation fragment (higher expression of the α_v_β_3_ integrin) had been used.

### 2.8. In Vitro Specific Binding to C6 Cells

**Figure 8:**
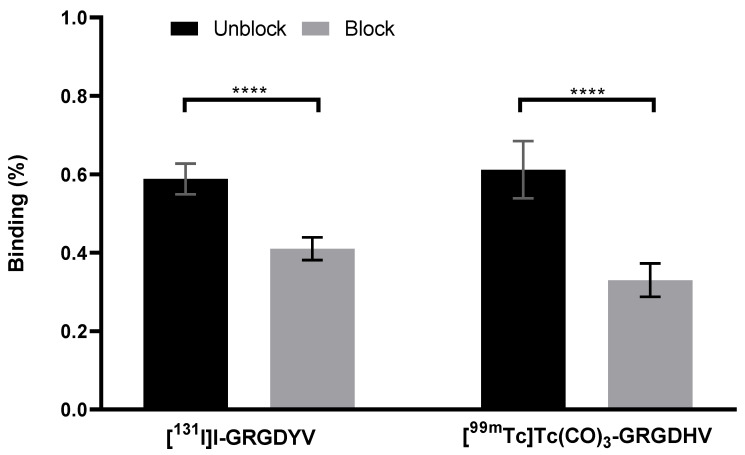
Specific binding of radiolabeled peptides to C6 cells. Data are expressed as ‘mean ± SD’. *p* < 0.0001 (****).

In vitro specific binding assays showed that the excess of the non-labeled peptide was able to decrease the binding percentage of the radiopeptide to C6 cells, as shown in Figure 8.

The [^131^I]I-GRGDYV fragment showed binding of 0.58 ± 0.39% and a reduction of ~30% (0.41 ± 0.02%) when incubated with an excess of non-radiolabeled GRGDYV fragment. A decrease was also observed for the [^99m^Tc]Tc(CO) _3_-GRGDHV complex (~46%) when incubated with an excess of non-radiolabeled GRGDHV fragment (0.61 ± 0.07% versus 0.33 ± 0.04). Therefore, these data indicate the binding specificity of the radiopeptides to C6 cells, once the excess of the non-radiolabeled peptide was able to block the α_v_β_3_ integrin receptors.

### 2.9. Biodistribution Assessment

The biodistribution data are shown in Table 1. The wide uptake by the kidneys is an indication of the predominant renal excretion, for both radiopeptides. These data reinforce the logP values, showing that both radiopeptides have hydrophilic properties and, therefore, are excreted in the urine.

Significant differences were observed for brain uptake between the model and control groups for both radiopeptides, at all investigated time points (*p* < 0.001). Higher accumulation in the brain of glioblastoma allograft tumor-bearing rats was observed when compared to control rats. Additionally, tumor brain uptake increased within time for both peptides and the accumulation of the [^131^I]I-GRGDYV (1.94 ± 0.02; 1.12 ± 0.03; 2.43 ± 0.43) in the brain was higher than that observed for the [^99m^Tc]Tc(CO)_3_-GRGDHV (0.71 ± 0.01; 0.81 ± 0.01; 1.57 ± 0.11). This difference suggests that the presence of the tricarbonyl group was able to alter the uptake by the tumor. These data might be explained by the higher SPB of this analog when compared to the ^131^I-labeled one. The higher tumor brain uptake can be explained by the overexpression of α_v_β_3_ integrin adhesion molecules in glioblastoma [21].

However, biodistribution data of [^131^I]I-GRGDYV was not as consistent as those obtained for [^99m^Tc]Tc(CO)_3_-GRGDHV. The analysis of the values in Table 1 show that the ^131^I-labeled peptide presents considerable differences between the model and control groups, as can be observed by the uptakes in the evaluated organs, at all investigated time points. Furthermore, the high uptake of [^131^I]I-GRGDYV by the stomach suggests in vivo radiochemical instability, which leads to radioisotope detachment of the peptide. Meanwhile, brain data indicate that the intact fraction of the radiopeptide was able to differentiate between normal and glioblastoma-bearing brains. On the other hand, biodistribution data of the [^99m^Tc]Tc(CO)_3_-labeled peptides are more homogeneous between model and control groups. Besides that, the values indicate in vivo radiochemical stability.

Therefore, the presented results indicate that the peptides may differentiate between the normal and glioblastoma models. However, due to a better in vivo radiochemical stability, the [^99m^Tc]Tc(CO)_3_-GRGDHV present greater potential as an imaging tracer for glioblastoma. Prospective imaging studies need to be performed to confirm this hypothesis.

In summary, all data showed that the GRGDYV and GRGDHV analogs were efficiently synthesized and radiolabeled with ^131^I and [^99m^Tc][Tc(CO)_3_]^+^, respectively, showing radiochemical yields >95% and high radiochemical stability in saline solution and human serum up to 24 h. Both radiopeptides showed hydrophilic characteristics and SPB <50%. The binding and internalization studies showed good interaction with C6 glioma cells, as well as with brain homogenates derived from allograft glioblastoma tumor-bearing rats. Finally, ex vivo biodistribution studies indicate that [^99m^Tc]Tc(CO)_3_-GRGDHV is more radiochemical stable than the [^131^I]I-GRGDYV, and showed uptake of both radiopeptides by the brain with tumor.

## 3. Materials and Methods

The Wang resin and amino acid derivatives were purchased from Bachem (Torrance, CA, USA). All solvents were HPLC grade, and the reagents met Airman Certification Standards (ACS). The ^131^I and ^99m^Tc radionuclides were acquired from Nordion (Ottawa, ON, Canada) and distributed by the Energy and Nuclear Research Institute (IPEN-CNEN; Sao Paulo, SP, Brazil).

### 3.1. Peptide Synthesis

Peptides were synthesized manually employing the classical Fmoc-chemistry methodology [28]. The α-amino group deprotection procedures were carried out using a solution containing 20% 4-methyl-piperidine in dimethylformamide (DMF) with stirring for 20 min. Coupling reactions were carried out using 2.5 excess of Fmoc-amino acid in the presence of 2-(1*H*-benzotriazol-1-yl)-1,1,3,3-tetramethyluronium tetrafluoroborate (TBTU)/diisopropylethylamine (DIEA) in a 1:1 (*v*/*v*) dichloromethane (DCM)/DMF mixture as a solvent system. After a 2 h period, the coupling reactions were qualitatively proven by the Kaiser test [29]. To check the yield and purity of the synthesized peptide sequences, cleavage reactions were performed in acid solution (Reagent K). The peptides (GRGDYV and GRGDHV) were synthesized at the 1.0 mmol scale on Wang resin (0.70 mmol·g^−1^).

### 3.2. RP-HPLC Analyses

RP-HPLC analyzes were performed on a Proeminence LC-20A system (Shimadzu; Kyoto, Japan). The column selected was Vydac C18 150 × 4.6 mm, with a particle size of 5 μm, a pore size of 300 Å. Chromatograms were performed at λ = 220 nm. Peaks were eluted using solvent systems composed of 0.1% TFA:H_2_O as solvent A and 60% acetonitrile/0.1% TFA:H_2_O as solvent B, on a gradient of 5–95% of solvent B at a flow rate of 1.0 mL·min^−1^ for 30 min (*n* = 3).

### 3.3. Preparative RP-HPLC

Purification of the peptide compounds was performed using 0.1% TFA:H_2_O as solvent A and 60% acetonitrile/0.1% TFA:H_2_O as solvent B. Considering the R_t_ determined by analytical chromatography and the concentration of solvent B in which the peptide was eluted, a linear gradient was applied. The flow was 10 mL·min^−1^ and peak detection was performed at 220 nm.

### 3.4. Liquid Chromatography-Electrospray Mass Spectrometry (LC/ESI-MS)

Liquid chromatography-mass spectrometry (LC/MS) was developed on a Waters Alliance model 2690 system and model 996 photodiode array detector (Waters; Eschborn, Germany) controlled by a Micromass model ZMD mass detector (Micromass; Altrincham, UK). Peptide samples were applied to a 150 × 2.1 mm C18 column, 3.5 µm particle size, 60 Å pore size (Nova-Pak, Waters, Milford, MA, USA). Elution was performed with 0.1% TFA:H_2_O as solvent A and 60% acetonitrile:0.1% TFA:H_2_O as solvent B, at a flow rate of 0.4 mL·min^−1^, using a linear gradient from 5% to 95% for solvent B for 30 min at 220 nm and a mass range of 500–3930 Da.

### 3.5. Radiolabeling of Peptides

Radiolabeling processes were optimized to produce radiolabeled peptides with high radiochemical yield.

For the peptide fragment GRGDYV, the radiolabeling process was performed with ^131^I radionuclide and the radioiodination was performed using the chloramine T methodology [20]. Experimentally, in a vial containing 25 µg (37 nmol) of peptide in 0.1 M phosphate buffered saline (PBS), with pH 7.3, 12 MBq (for in vitro studies) and 25 MBq (for in vivo studies) of Na^131^I and 5 μL of Chloramine T (1 mg·mL^−1^) were incorporated. After 90 s at room temperature, an aliquot of 5 μL of sodium metabisulfite (2 mg·mL^−1^) was added to finish the reaction.

For the analog GRGDHV (containing the histidine amino acid), the radiolabeling was realized with the [^99m^Tc][Tc(CO)_3_]^+^ complex following the protocol described by Waibel et al. [19] and involves the following steps:

(i) Synthesis of [^99m^Tc(H_2_O)_3_(CO)_3_]^+^ core:

Carbon monoxide gas was purged through an aqueous solution of NaBH_4_ (5 mg), Na_2_CO_3_ (8 mg), and Na/K tartrate (18 mg) in 0.5 mL of distilled water for 10 min. To this solution, freshly eluted Na^99m^TcO_4_ (1 mL, ~37 MBq) was added, and the reaction mixture was incubated at 75 °C for 35 min. The pH of the solution was adjusted to 7.0 using 0.1 M HCl.

(ii) Labeling of histidine of the peptide sequence with the [^99m^Tc][Tc(CO)_3_]^+^:

The radiolabeling reaction was performed with 250 µg (39 nmol) of the His-containing peptide mixed with 200 µL of [^99m^Tc][Tc(CO)_3_]^+^ solution previously prepared [19] with activity of 370 MBq (for in vitro studies) or 630 MBq (for in vivo studies). In both cases, the mixture was heated at 37 °C for 90 min.

### 3.6. Radiochemical Yield

The radiolabeling yield of [^131^I]I-peptide was determined by TLC using Whatmann 3 MM strips and MeOH/H_2_O (95:5), as eluent. For [^99m^Tc]Tc(CO)_3_-peptide, TLC-SG (Al) strips (Merck; Burlington, MA, USA) using ACN/H_2_O (95:5) as eluent were used; and for only [^99m^Tc][Tc(CO)_3_]^+^ complex the radiolabeling yield was evaluated using TLC-SG (Al) strips and 0.9% NaCl, as eluent. The radioactivity was determined by AR 2000 TLC-Scanner (Eckert & Ziegler; Berlin, Germany). 

RP-HPLC analyzes were performed on a 1290 Infinity II UHPLC system (Agilent Technologies; Saint Clair, CA, USA) fitted with a radioactivity detector (Eckert & Ziegler; Berlin, Germany) and Open Lab ECM data system (Agilent Technologies; Saint Clair, CA, USA) to confirm the yield and purity of both radiolabeled peptides. Analyzes were performed using a Phenomenex C18 column (150 × 46 mm; 5 µm particle size; 300 Å pore size), detection at λ = 220 nm, using 0.1% TFA:H_2_O as solvent A and 60% acetonitrile/0.1% TFA:H_2_O as solvent B. A gradient of 5% to 95% of solvent B in 30 min was used at a flow rate of 1.0 mL·min^−1^.

### 3.7. In Vitro Stability Studies

In vitro stability studies were performed in saline, under refrigeration (4–8 °C) and at room temperature, for up to 24 h after radiolabeling (*n* = 6). Stability studies were also conducted in serum, after incubation at 37 °C for up to 24 h. In all cases, samples were evaluated by TLC, as described above.

### 3.8. Partition Coefficient (P) Determination

A fraction (100 μL) of the radiopeptide was added to a water/n-octanol (1:1) mixture. The mixture was vigorously vortexed and kept at rest for 1 h. Then, it was centrifuged at 1677× *g* for 5 min. Equal samples from both aqueous/organic phases were collected and their radioactivities were measured in an automatic Wizard 3 2480 gamma counter (Perkin Elmer; Norwalk, CT, USA) to determine log *P* values (*n* = 5).

### 3.9. Serum Protein Binding

Radiopeptides (25 μL) were added to 175 µL of serum (*n* = 6) and incubated at 37 °C for 1 h. After, 1 mL of 10% trichloroacetic acid (TCA) was added for the precipitation of proteins. The mixture was vortexed for 1 min and centrifuged at 1677× *g* for 10 min. This process was repeated three times. Both the supernatant’s and pellet’s radioactivity were measured in an automatic Wizard 3 2480 gamma counter (Perkin Elmer; Norwalk, CT, USA).

### 3.10. C6 Glioblastoma Cell Culture

The C6 cells were incubated at 37 °C in a humidified atmosphere containing 5% CO_2_ and maintained in culture using Dulbecco’s Modified Eagle Medium (DMEM)—F12 (Gibco; Gaithersburg, MD, USA) supplemented with 10% fetal bovine serum (Sigma-Aldrich; Darmstadt, Germany) and 1% glutamine (Sigma-Aldrich; Darmstadt, Germany). Cells were grown to 75% confluence and then harvested by trypsinization (0.04% trypsin/EDTA). After centrifugation (151× *g* for 5 min), the cells were resuspended in DMEM—F12 to a final concentration of 10^6^ cells/µL for the development of the glioblastoma animal model.

### 3.11. Binding and Internalization Studies of Radioconjugates in C6 Tumorigenic Cells 

An aliquot containing 2 × 10^6^ C6 cells in supplemented DMEM-F12 culture medium (450 µL) was added to a vial and incubated with 10 nmol of the radiolabeled peptide at 37 °C with gentle agitation. The studies were evaluated at 1, 4 and 24 h (*n* = 7 for each time point). Then, the vials were centrifuged (604× *g* for 5 min). The radioactivities of the pellet and supernatant were separately evaluated using an automatic Wizard 3 2480 gamma counter (Perkin Elmer; Norwalk, CT, USA). The percentage of the bound fraction was calculated by Equation (1).

(1) $$\%\;bound\;fraction=\frac{Pellet\;radioactivity\;\left(cpm\right)}{Pellet\;radioactivity\;\left(cpm\right)+supernatant\;radioactivity\;\left(cpm\right)}\times 100$$

In the sequence, a second radioactivity determination was performed. For that, pellets were resuspended using 0.5 mL of acid buffer (0.2 M acetic acid in 0.5 M NaCl solution; pH 2.8) and maintained at room temperature for 5 min to remove the radiolabeled molecule attached to the cell membrane surface. Then, the vials were centrifuged (1677× *g* for 5 min). The pellet and supernatant were separated and their radioactivities were measured using the above-mentioned automatic gamma counter. The percentage of the internalized fraction of radiopeptides was estimated based on the ratio between the activity contained in the precipitate and the supernatant and calculated by Equation (2):

(2) $$\%\;internalized\;fraction=\frac{Pellet\;radioactivity\;\left(cpm\right)}{Pellet\;radioactivity\;\left(cpm\right)+supernatant\;radioactivity\;\left(cpm\right)}\times 100$$

### 3.12. Glioblastoma Allograft Tumor-Bearing Animal Model

The animals used in this experiment were provided by the vivarium of our institution, which is certified by the International Association for the Assessment and Accreditation of Laboratory Animal Care (AAALAC). Every effort was taken to minimize animal suffering in accordance with International Ethical Guidelines. The experimental protocols used in this study were approved by the Scientific Ethics Committee of the Hospital Israelita Albert Einstein.

Adult male Sprague Dawley rats (280–320 g) were maintained under controlled environmental conditions (12 h light-dark cycle; constant temperature 21 ± 2 °C), with free access to water and food. Tumorigenic glioblastoma cells were implanted in the right frontal cortex, following specific coordinates (referred to bregma): anteroposterior = 1.0 mm; lateral = 3.0 mm; depth = 5.0 mm [30]. An aliquot of culture medium (10 µL) containing 1 × 10^6^ C6 cells in suspension was injected slowly (1 µL/min) using a Hamilton syringe positioned on a stereotaxic surgery frame. In the control group, only the culture medium, without cells, was injected. An interval of 20 days after cell inoculation was allowed for tumor development.

### 3.13. Binding and Internalization Studies of Radioconjugates in Brain Homogenate

Brain homogenates were prepared at the concentration of ~10 mg of wet tissue in 1000 µL of PBS (pH 7.4). Normal brain tissue (control) and tumor-bearing brain tissue (model) were used. A 25 µL aliquot (1.2 MBq/nmol) of each radiopeptide was added to 200 µL of freshly prepared homogenates (*n* = 7). The homogenates containing the radiolabeled peptides were incubated at 37 °C for 1, 4 and 24 h. Binding and internalization fractions were determined as described in Section 3.11.

### 3.14. In Vitro Specific Binding to C6 Cells

The specific binding of the radiopeptides to C6 cells was evaluated in 12-well adherent plates, containing 2.0 × 10^5^ cells per well. For the specific binding study (unblock), supplemented DMEM-F12 medium containing 1.66 nmol of the [^131^I]I-GRGDYV or 8.88 nmol of the [^99m^Tc]Tc(CO) _3_-GRGDHV was added in the wells. On the other hand, for the non-specific binding study (block), an excess of 1000× of the respective non-radiolabeled peptide (competitor) was added to the supplemented DMEM-F12 medium containing the radiopeptide in each well. In both cases, the plates were incubated at 37 °C in a humidified atmosphere containing 5% CO_2_ for 4 h and then, the supernatant was removed. Each well was washed with PBS, which was joined to the respective supernatant. Finally, the cells were removed by trypsinization and the contents were placed in microtubes. Each well was washed again with PBS, which was joined to the respective trypsinized cells. Cells and supernatants were counted in the above-mentioned automatic gamma counter.

### 3.15. Ex Vivo Biodistribution Studies

The ex vivo biodistribution of each radiolabeled peptide (4 MBq; 0.2 mL) was evaluated at 15, 60, and 240 min after IV injection in control and tumor-bearing animals (*n* = 3 per group, per time point). The molar activities in this study were 0.68 and 16.2 MBq/nmol for the iodine and tricarbonyl-labeled peptides, respectively. Animals were deeply anesthetized and sacrificed at the corresponding biodistribution time point. Brain, spleen, heart, stomach, liver, lungs, kidneys, and blood were collected and weighed. Then, their radioactivities were determined using the above-mentioned automatic gamma counter. The radioactivity of each organ/tissue was evaluated as the percentage of injected dose per gram of organ/tissue (% ID/g), using the same injected dose activity as a standard.

### 3.16. Statistical Analysis

The Student’s *t*-test was used to compare the means of two groups and Analysis of Variance (ANOVA), followed by the Tukey post-test, was used for three groups. A significance interval of 95% was considered (*p*-value < 0.05). The data were analyzed using the GraphPad Prism v.8.0.2 software (GraphPad Software Inc., La Joya, CA, USA).

## 4. Conclusions

In conclusion, the radiolabeling efficiency and the radiochemical stability of both radiopeptides were quite similar, but the exchange of radionuclides and the presence of a tricarbonyl group altered the physicochemical features of peptides and their in vitro interactions with glioma cells, as well as the peptides biodistribution. Although the brain uptake of both radiopeptides was higher in glioblastoma tumor-bearing rats, when compared to normal animals, the ^131^I-labeled peptide showed low in vivo radiochemical stability. Our data suggest that the [^99m^Tc][Tc(CO)_3_]-labeled peptide has the most interesting and consistent characteristics, with the potential to be used in the future as a radiopharmaceutical for glioblastoma by SPECT imaging.

## Figures and Tables

**Table 1:** Ex vivo biodistribution data obtained after intravenous injection of [^131^I]I-GRGDYV and [^99m^Tc]Tc-GRGDHV into glioblastoma allograft tumor-bearing (model) and normal (control) rats (*n* = 3 per group, per time).

Organ/Tissue	15 min		60 min		240 min	
	Model	Control	Model	Control	Model	Control
	**[^131^I]I-GRGDYV**					
**Brain**	1.94 ± 0.02 ***	**0.31 ± 0.04**	1.12 ± 0.03 ***	**0.34 ± 0.06**	2.43 ± 0.43 ***	**0.33 ± 0.04**
Spleen	6.99 ± 0.13	3.65 ± 0.49	4.94 ± 0.76	2.60 ± 0.10	2.14 ± 0.36	3.39 ± 1.31
Heart	8.23 ± 0.48	1.60 ± 0.33	5.54 ± 0.37	0.49 ± 0.10	2.74 ± 0.66	0.84 ± 0.70
Stomach	33.05 ± 2.45	72.19 ± 0.61	55.93 ± 1.82	70.67 ± 0.39	3.59 ± 0.46	0.18 ± 0.03
Liver	8.93 ± 0.53	1.33 ± 0.05	5.13 ± 0.52	0.27 ± 0.01	2.75 ± 1.64	0.85 ± 0.64
Lungs	11.96 ± 1.02	22.57 ± 0.62	8.27 ± 0.09	6.99 ± 1.11	4.00 ± 0.46	2.90 ± 0.64
Kidneys	15.83 ± 0.96	49.24 ± 1.99	9.14 ± 0.69	57.14 ± 2.19	79.19 ± 1.63	77.11 ± 1.86
Blood	15.15 ± 0.59	5.42 ± 0.72	10.91 ± 0.32	28.83 ± 2.36	4.46 ± 0.40	7.04 ± 2.28
		**[^99m^Tc]Tc(CO)_3_-GRGDHV**				
**Brain**	0.71 ± 0.01 ***	**0.32 ± 0.01**	0.81 ± 0.01 ***	**0.28 ± 0.03**	1.57 ± 0.11 ***	**0.60 ± 0.04**
Spleen	3.74 ± 0.33	3.41 ± 0.41	2.88 ± 0.32	3.69 ± 0.60	4.12 ± 0.79	4.93 ± 0.45
Heart	5.40 ± 0.48	5.14 ± 0.12	5.14 ± 0.73	4.41 ± 0.23	4.80 ± 0.11	4.84 ± 0.37
Stomach	4.44 ± 1.25	3.09 ± 0.77	7.28 ± 0.88	10.27 ± 0.59	14.60 ± 2.07	10.78 ± 3.11
Liver	7.04 ± 0.68	6.06 ± 0.79	8.29 ± 0.43	9.55 ± 1.34	11.62 ± 1.01	12.26 ± 1.34
Lungs	10.03 ± 2.56	7.76 ± 0.75	7.83 ± 0.20	9.25 ± 1.65	9.38 ± 1.89	8.59 ± 0.64
Kidneys	58.33 ± 1.43	62.73 ± 2.17	58.99 ± 1.13	51.04 ± 1.49	40.44 ± 2.99	40.17 ± 0.90
Blood	10.28 ± 2.93	11.30 ± 0.84	8.91 ± 0.94	9.78 ± 0.52	10.32 ± 1.51	11.12 ± 1.27

Data are represented as the percentage of the injected dose per gram of tissue (%ID/g) and are expressed as ‘mean ± SEM’. Asterisks indicate significant differences between model and control groups in the respective biodistribution time point for brain data (*** *p* < 0.001).

## Data Availability

The data presented in this study are available on request from the corresponding author. The data are not publicly available due to privacy.

## References

[1] Blom E., Velikyan I., Estrada S., Hall H., Muhammad T., Ding C., Nair M., Långström B. (2012). ^68^Ga-Labeling of RGD peptides and biodistribution. Int. J. Clin. Exp. Med..

[2] Folkman J. (1997). Angiogenesis and angiogenesis inhibition: An overview. EXS.

[3] Niu G., Chen X. (2011). Why Integrin as a Primary Target for Imaging and Therapy. Theranostics.

[4] Oliveira M.C., Correia J.D.G. (2019). Biomedical applications of radioiodinated peptides. Eur. J. Med. Chem..

[5] Vats K., Agrawal K., Sharma R., Sarma H.D., Satpati D., Dash A. (2019). Preparation and clinical translation of ^99m^Tc-PSMA-11 for SPECT imaging of prostate cancer. MedChemComm.

[6] Laubenstein M., Lawson I. (2020). Low Background Radiation Detection Techniques and Mitigation of Radioactive Backgrounds. Front. Phys..

[7] Shi J., Wang F., Liu S. (2016). Radiolabeled cyclic RGD peptides as radiotracers for tumor imaging. Biophys. Rep..

[8] Macdonald T.J., Ladisch S. (2001). Antisense to integrin alpha v inhibits growth and induces apoptosis in medulloblastoma cells. Anticancer Res..

[9] MacDonald T.J., Taga T., Shimada H., Tabrizi P., Zlokovic B.V., Cheresh D.A., Laug W.E. (2001). Preferential susceptibility of brain tumors to the antiangiogenic effects of an alpha(v) integrin antagonist. Neurosurgery.

[10] Chen H., Niu G., Wu H., Chen X. (2016). Clinical Application of Radiolabeled RGD Peptides for PET Imaging of Integrin α_v_β_3_. Theranostics.

[11] Chatterjee S., Matsumura A., Schradermeier J., Gillespie G.Y. (2000). Human malignant glioma therapy using anti-αVβ_3_ integrin agents. J. Neurooncol..

[12] Taga T., Suzuki A., Gonzalez-Gomez I., Gilles F.H., Stins M., Shimada H., Barsky L., Weinberg K.I., Laug W.E. (2002). αv-Integrin antagonist EMD 121974 induces apoptosis in brain tumor cells growing on vitronectin and tenascin. Int. J. Cancer.

[13] Malavolta L., Cabral F.R. (2011). Peptides: Important tools for the treatment of central nervous system disorders. Neuropeptides.

[14] Carter A. (2010). Integrins as Target: First Phase III Trial Launches, but Questions Remain. J. Natl. Cancer Inst..

[15] Liu S. (2006). Radiolabeled multimeric cyclic RGD peptides as integrin α_v_β_3_ targeted radiotracers for tumor imaging. Mol. Pharm..

[16] Pirooznia N., Abdi K., Beiki D., Emami F., Arab S.S., Sabzevari O., Pakdin-Parizi Z., Geramifar P. (2020). Radiosynthesis, Biological Evaluation, and Preclinical Study of a ^68^Ga-Labeled Cyclic RGD Peptide as an Early Diagnostic Agent for Overexpressed α_v_β_3_ Integrin Receptors in Non-Small-Cell Lung Cancer. Contrast Media Mol. Imaging.

[17] Novy Z., Stepankova J., Hola M., Flasarova D., Popper M., Petrik M. (2019). Preclinical Evaluation of Radiolabeled Peptides for PET Imaging of Glioblastoma Multiforme. Molecules.

[18] Ferris T., Carroll L., Jenner S., Aboagye E.O. (2021). Use of radioiodine in nuclear medicine—A brief overview. J. Label. Compd. Radiopharm..

[19] Waibel R., Alberto R., Willuda J., Finnern R., Schibli R., Stichelberger A., Egli A., Abram U., Mach J.-P., Plückthun A. (1999). Stable one-step technetium-99m labeling of His-tagged recombinant proteins with a novel Tc(I)–carbonyl complex. Nat. Biotechnol..

[20] Hunter W.M., Greenwood F.C. (1962). Preparation of iodine-131 labeled human growth hormone of high specific activity. Nature.

[21] Temming K., Schiffelers R., Molema G., Kok R.J. (2005). RGD-based strategies for selective delivery of therapeutics and imaging agents to the tumour vasculature. Drug Resist. Updates.

[22] de Barros A.L.B., Ferraz K.S.D.O., Dantas T.C.S., Andrade G.F., Cardoso V.N., de Sousa E.M.B. (2015). Synthesis, characterization, and biodistribution studies of ^99m^Tc-labeled SBA-16 mesoporous silica nanoparticles. Mater. Sci. Eng. C.

[23] Brunton L., Parker K., Blumenthal D., Buxton I. (2005). Goodman & Gilman: Manual e Farmacologia e Terapêutica.

[24] Croom E. (2012). Metabolism of Xenobiotics of Human Environments. Prog. Mol. Biol. Transl. Sci..

[25] Oliveira E.A., Faintuch B.L. (2015). Radiolabeling and biological evaluation of the GX1 and RGD-GX1 peptide sequence for angiogenesis targeting. Nucl. Med. Biol..

[26] Sobral D.V., Fuscaldi L.L., Durante A.C.R., Rangel M.G., Oliveira L.R., Mendonça F.F., Miranda A.C.C., Cabeza J.M., Montor W.R., Cabral F.R. (2020). Radiochemical and biological properties of peptides designed to interact with EGF receptor: Relevance for glioblastoma. Nucl. Med. Biol..

[27] Fuscaldi L.L., Sobral D.V., Durante A.C.R., Mendonça F.F., Miranda A.C.C., da Cunha M.L., Malavolta L., Mejia J., de Barboza M.F. (2021). Standardization of the [^68^Ga]Ga-PSMA-11 Radiolabeling Protocol in an Automatic Synthesis Module: Assessments for PET Imaging of Prostate Cancer. Pharmaceuticals.

[28] Fields G.B., Noble R.L. (1990). Solid phase peptide synthesis utilizing 9-fluorenylmethoxycarbonyl amino acids. Int. J. Pept. Protein Res..

[29] Kaiser E., Colescott R.L., Bossinger C.D., Cook P.I. (1970). Color test for detection of free terminal amino groups in the solid-phase synthesis of peptides. Anal. Biochem..

[30] Zhang J., Liu H., Du X., Guo Y., Chen X., Wang S., Fang J., Cao P., Zhang B., Zhang W. (2017). Increasing of Blood-Brain Tumor Barrier Permeability through Transcellular and Paracellular Pathways by Microbubble-Enhanced Diagnostic Ultrasound in a C6 Glioma Model. Front. Neurosci..

